# Recycled or Bio-Based Solvents for the Synthesis of ZnO Nanoparticles: Characterization and Validation in Organic Solar Cells

**DOI:** 10.3390/ma17061332

**Published:** 2024-03-14

**Authors:** Cristiano Albonetti, Riva Alkarsifi, Virginie El Qacemi, Benjamin Dhuiege, Giampiero Ruani, Mirko Seri

**Affiliations:** 1 National Research Council (CNR)—Institute of Nanostructured Materials (ISMN), Via P. Gobetti 101, 40129 Bologna, Italy; 2 GenesInk, 39 Avenue Gaston Imbert, 13790 Rousset, France

**Keywords:** ZnO nanoparticles, recycled or bio-based solvents, solution processing, electron-transporting layer, organic solar cells

## Abstract

Among solution-processable metal oxides, zinc oxide (ZnO) nanoparticle inks are widely used in inverted organic solar cells for the preparation, at relatively low temperatures (<120 °C), of highly efficient electron-transporting layers. There is, however, a recent interest to develop more sustainable and less impacting methods/strategies for the preparation of ZnO NPs with controlled properties and improved performance. To this end, we report here the synthesis and characterization of ZnO NPs obtained using alternative reaction solvents derived from renewable or recycled sources. In detail, we use (i) recycled methanol (*r*-MeOH) to close the loop and minimize wastes or (ii) bioethanol (*b*-EtOH) to prove the effectiveness of a bio-based solvent. The effect of *r*-MeOH and *b*-EtOH on the optical, morphological, and electronic properties of the resulting ZnO NPs, both in solution and thin-films, is investigated, discussed, and compared to an analogous reference material. Moreover, to validate the properties of the resulting materials, we have prepared PTB7:PC_71_BM-based solar cells containing the different ZnO NPs as a cathode interlayer. Power conversion efficiencies comparable to the reference system (≈7%) were obtained, validating the proposed alternative and more sustainable approach.

## 1. Introduction

Metal oxides (MOs) have been extensively investigated for decades due to their exceptional characteristics, including adaptable electronic properties, excellent carrier mobilities, high optical transparency, mechanical stress tolerance, and compatibility with different classes of materials. High-quality electronic-grade MO thin-films can be deposited using vapor- or solution-phase techniques under mild conditions, thus widening their use in a variety of applications [1,2,3].

MO thin-films have been extensively used as interlayers, both as *p*-type and *n*-type materials, in new emerging organic electronic devices such as organic light-emitting diodes (OLEDs) and organic solar cells (OSCs), providing systems with improved performance and operational stability [4,5].

Among the various solution-processable MOs used for OSCs, ZnO is one of the well-known and consolidated electron-transporting layers (ETLs) due to its specific advantages such as low cost; minimal toxicity; easy solution processing; and suitable optical, electrical and electronic properties [6]. ZnO is mainly used as a cathode interlayer in inverted OSCs by directly depositing an ETL on the top of the Indium Tin Oxide (ITO), improving the electron extraction efficiency at the cathode [7]. 

To date, conventional methods of preparing ZnO films from solution-phase consist of the use of sol–gel processes or pre-synthesized ZnO nanoparticles (NPs). Sol–gel processing, based on the use of soluble precursors, is an effective way to generate ZnO films. However, it requires strong thermal treatments (T > 200 °C) to obtain high-performance thin-films [8]. The use of pre-synthesized ZnO NPs dispersed in solution is an alternative strategy for the deposition of ZnO thin-films under mild processing conditions (T < 100 °C), compatible with plastic (flexible) substrates [6]. Since the advent of ZnO NPs, researchers are still working on the optimization of material properties, ink formulation, and synthetic protocols to redefine the procedures, reduce costs, and facilitate processing without losing the level of performance [9]. However, up to now, little attention has been devoted to the sustainable development of this material and its related processes. Recently, there has been a growing interest for the preparation of green-synthesized ZnO NPs for a variety of applications [10,11,12], even including photovoltaic devices [13,14,15]. Therefore, the possibility to develop alternative and less impacting methods/strategies to prepare ZnO NPs with controlled properties and improved performance is of relevant importance for the design of the next generation of more sustainable and efficient materials/components and devices [16,17,18,19].

In this work, we propose an innovative and more sustainable synthetic approach for the preparation of ZnO NPs by using alternative solvents of synthesis derived from renewable or recycled sources. The synthesis of ZnO NPs was commonly carried out in methanol (MeOH) [20,21], a solvent that limits the environmental performance of the resulting materials as it is toxic, flammable and derived from fossil resources. Therefore, we report here two alternative strategies to minimize or eliminate the use of MeOH. In detail, we used (i) recycled methanol (*r*-MeOH) to close the loop and minimize its use or (ii) bioethanol (*b*-EtOH) to prove the feasibility of replacing methanol with a less impacting bio-based solvent. 

The effect of *r*-MeOH and *b*-EtOH as reaction solvents on the optical, morphological, and electronic properties of the resulting ZnO NPs, both in solution and thin-films, was investigated, discussed and compared to an analogous reference material obtained from commercial (non-recycled) MeOH. Moreover, to evaluate the electrical performance of the resulting thin-films, we fabricated a set of inverted OSCs using ZnO NPs as an ETL and PTB7:PC_71_BM as a photoactive blend. The resulting devices exhibited power conversion efficiencies (PCEs) between 6.5 and 6.9%, consistent with analogous systems reported in the literature [22,23], thus validating the effectiveness of the proposed synthetic procedures.

## 2. Materials and Methods

### 2.1. Materials

Zinc acetate dihydrate (99.8%), potassium hydroxide (85%) and ethanol absolute ≥99.5% Ph. Eur., USP (bioethanol, obtained from vegetal origin), were purchased from VWR and used without further treatments. Further details/characteristics on bioethanol can be found on the website and related documentation [24]. Anhydrous methanol (≥99.8%) was purchased from Sigma-Aldrich and used without further purification. The acceptor material, PC_71_BM ([6,6]-phenyl-C71-butyric acid methyl ester), was purchased from Solenne BV, while the donor polymer, PTB7 (Poly [[4,8-bis[(2-ethylhexyl)oxy]benzo [1,2-b:4,5-b′]dithiophene-2,6-diyl][3-fluoro-2-[(2-ethylhexyl)carbonyl] thieno [3,4-b] thiophenediyl]]), was purchased from American Dye. The MoO_x_ and Ag were purchased from Sigma-Aldrich and K.J. Lesker, respectively, and used without additional treatments and/or purifications.

### 2.2. Preparation of the ZnO NP Inks

#### 2.2.1. Synthesis of the Ref-ZnO NPs

A total of 0.3 mol/L of KOH in MeOH solution was added dropwise to 0.11 mol/L of zinc acetate dihydrate dissolved in MeOH. The mixture was clear and colorless at the end of the addition. After stirring for 135 min at 60 °C, the medium turned opaque white (milky appearance), and the reaction was left to settle at room temperature overnight. The ZnO NPs were purified up to 5 times by successive washing with methanol. For the preparation of 1% *wt*/*v* ZnO inks, the NPs were dispersed in an alcohol-based solvent with <10% *wt*/*v* of the ligand (ink formulation protected by patent [25]).

#### 2.2.2. Synthesis of r-ZnO NPs

For the synthesis and preparation of *r*-ZnO NPs (and ink), we used the same conditions and procedures adopted for the Ref-ZnO NPs, as reported above in Section 2.2.1.

Methanol recycling: The methanol used for recycling was previously employed in the typical ZnO synthesis washing step; as a result, synthesis byproducts, potassium acetate and excess starting materials, were likely sources of contamination. For recycling, methanol was mixed with dry CaO and distilled at 700 rpm under argon. The CaO was used as a drying agent to trap water in the solvent medium. The mixture was left under heating in a closed circuit for 25 min from the start of reflux. Recycled methanol was collected for approximately 3 h with a yield of 80%.

#### 2.2.3. Synthesis of b-ZnO NPs

The same procedure was followed for the synthesis of *b*-ZnO in bio-based ethanol instead of methanol. The only difference was the increased temperature from 60 °C to 80 °C, while the reaction time remained the same. For the preparation of 1% *wt*/*v* ZnO inks, the NPs were dispersed in an alcohol-based solvent with <10% *wt*/*v* of the ligand (ink formulation protected by patent [25]).

### 2.3. Characterization Methods

The NP size distribution within the solution was determined by DLS using a Nanosizer from Malvern (Worcestershire, UK). Thermogravimetric analysis (TGA) was performed on a TGA Q50 instrument (TA Instruments, New Castle, PA, USA) in air with a temperature scanning rate of 10 °C·min^−1^ from RT to 600 °C. The viscosity was measured using a Rheometer ARG2 (TA Instruments, New Castle). The UV–vis spectra of the ZnO NPs in solution were recorded at room temperature using an Agilent Cary 100 spectrophotometer (Agilent, Santa Clara, CA, USA).

The optical properties of the ZnO thin-films were measured using a JASCO V-550 spectrophotometer (Jasco, Court Easton, MD, USA). The thin-films were prepared by blade coating, using the same conditions employed for device fabrication. The thicknesses of the resulting films were measured using a profilometer (Tencor P-6 profilometer, KLA, Leuven, Belgium).

Infrared spectra of the ZnO films, deposited on glass/ITO, were acquired using the attenuated total reflectance technique (ATR) with a Vertex 70 spectrometer (Bruker, Billerica, MA, USA) equipped with a diamond crystal single-reflection Platinum ATR accessory, in the 400–4000 cm^−1^ region, with 128 scans and a resolution of 4 cm^−1^.

Topographic AFM images were collected using a SOLVER HV-MFM microscope (NT-MDT, Zelenograd, Moscow, Russia), operating in Intermittent Contact Mode with HA_NC Etalon cantilevers (resonant frequencies ω_0_ of ≈140 and ≈240 kHz and elastic constants *k* of ≈4 and ≈12 N·m^−1^). Surface potential images were obtained by KPFM on a SOLVER P47 PRO (NT-MDT, Zelenograd, Moscow, Russia), using NSG11 Pt-coated cantilevers (ω_0_ ≈ 150 kHz and *k* of ≈6 N·m^−1^).

### 2.4. Device Fabrication

The device architecture comprised glass/ITO/ZnO/active blend/MoO_x_/Ag. In agreement with reference [16], patterned ITO-coated glasses (Rs~10 Ω/square) were cleaned in sequential sonicating baths (for 15 min) in deionized water, acetone, and isopropanol. After the final sonication step, substrates were dried with a stream of Ar gas and then placed in an oxygen plasma chamber for 5 min. ITO (Indium Tin Oxide) is a transparent conductive oxide deposited as a thin layer (≈150 nm) on the top of a glass substrate to provide conductivity. The glass/ITO is the conventional and widely used (conductive) substrate for the fabrication of organic electronic devices.

A thin layer of ZnO (~50 nm) was blade-coated in air (blade speed: 2.5 mm/s; height of the blade: 100 μm; volume: 45 μL; hot-plate temperature: 30 °C) on the top of the glass/ITO substrates. The ZnO films were thermally annealed on a hot-plate at 120 °C for 10 min (or 85 °C for 20 min). The active blend solution was prepared as follows: PTB7:PC_71_BM (1:1.5 *wt*/*wt*) dissolved in CB + 3% (*v*/*v*) of 1,8-diiodooctane (DIO) with a total concentration of 25 mg/mL kept under stirring overnight at 65 °C. After dissolution, the active blend was spin-coated (in air) on the top of glass/ITO/ZnO at 1300 rpm for 120 s, without the need of additional thermal treatments. The films were then transferred inside a metal evaporator to complete the devices by depositing MoOx (10 nm) and Ag (100 nm) layers. The current–voltage (I-V) characteristics of all the solar cells were recorded using a Keithley 236 source measure unit under a simulated AM1.5G illumination of 100 mW/cm^2^ (Sun 2000 Solar Simulator, Abet Technologies, Milford, CT, USA)) inside a glove box. During photovoltaic characterization (under illumination), each cell was carefully masked with a calibrated mask with a spot of 6 mm^2^.

## 3. Results

### 3.1. ZnO Nanoparticle Inks

**Scheme 1:**
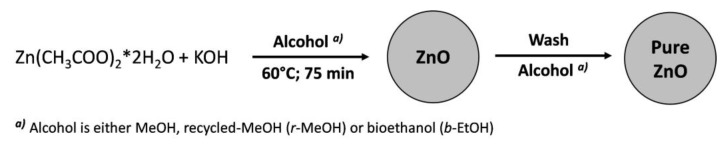
Schematic illustration of ZnO synthesis.

ZnO NPs were prepared using identical experimental conditions except for the solvent adopted in the synthesis (Scheme 1). Starting from zinc acetate dihydrate (precursor), Ref-ZnO was synthesized by using commercial methanol (MeOH), whereas *b*-ZnO and *r*-ZnO NPs were synthesized in bio-based ethanol (*b*-EtOH) and recycled methanol (*r*-MeOH), respectively. After the dropwise addition of the KOH–alcohol mixture, the reaction medium was stirred for ~2 h at 60 °C. The use of *b*-EtOH rather than MeOH required an increase in the reaction temperature from 60 to 80 °C to compensate for its lower solubility. After several purification steps, the synthesized NPs were redispersed in a commercial aliphatic monohydric alcohol with <10% (*wt*/*v*) ligand to hamper NP aggregation and obtain the final NP inks (ink formulation protected by patent) [25]. Further details on the material synthesis and characterization are reported in the Section 2.

The chemical and physical properties of the resulting NP inks were investigated in detail to evaluate the effect of the different reaction solvents.

**Figure 1:**
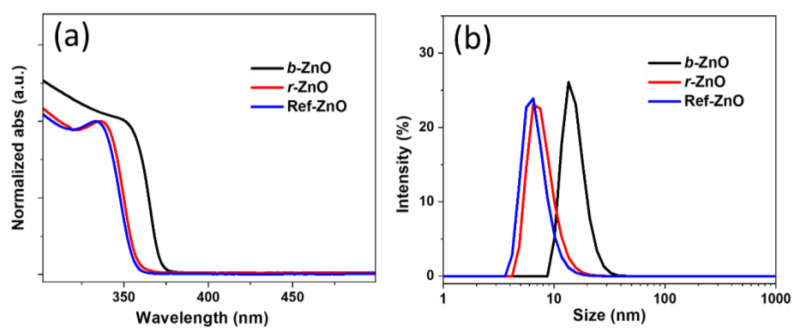
(**a**) UV–vis absorption spectra in solution and (**b**) DLS plots of the ZnO NP inks.

UV–vis optical absorption spectra of the inks show a strong absorption band in the UV region accompanied by a shoulder peak (λ*_sh_*) at relatively longer wavelengths (≈330–350 nm, Figure 1a), typical of ZnO NPs [26]. The different position of λ*_sh_* for the *b*-ZnO, *r*-ZnO and Ref-ZnO NP inks (≈350, 338 and 333 nm, respectively, see Table 1) is ascribable to the progressive reduction in particle size [26]. Indeed, decreasing the particle size increases the optical band gap, thus causing a blue-shift in the absorption wavelength (l*_sh_*). This effect can be explained and confirmed by several models that describe the complex correlation between the electronic structure (band gap) and size of the ZnO NPs [27].

The convolved shoulder peak observed for the *b*-ZnO NPs might be related to the effect of the different reaction solvent, which could influence not only the aggregation size but also some of the intrinsic properties of the NPs, such as slight changes to the degree of crystallinity, number of defects, etc., responsible for the observed features in the absorption spectra [21,28].

The dimension of the dispersed ZnO NPs was estimated by dynamic light scattering (DLS, Figure 1b). As reported in Table 1, the hydrodynamic diameters, *d*(*H*), were found to be ≈14, 7, and 6 nm for the *b*-ZnO, *r*-ZnO, and Ref-ZnO inks, respectively, following the same trend observed in the UV–vis analysis. The sharp intensity distributions of the DLS graphs indicate that all the inks contain monodispersed ZnO NPs. Comparable results were obtained from the DLS analysis carried out on the synthesized ZnO NPs (before redispersion, see Table 1 and Figure S1), highlighting the efficacy of the synthetic procedures for the generation of already small, monodispersed, and aggregate-free NPs.

The effective dimensions of the *r*-ZnO and *b*-ZnO NPs contained in the final inks were further investigated using transmission electron microscopy (TEM). As shown in Figure S2 (supporting information), the diameter measured from the TEM images, *d*(*T*), was (5 ± 1) and (6 ± 1) nm for the *b*-ZnO and *r*-ZnO NPs, respectively. They are equal within the experimental errors but smaller than the corresponding values estimated by DLS [29]. Within the experimental errors, the results of the TEM and DLS measurements are comparable for the *r*-ZnO NPs, and vice versa; the large difference between *d*(H) and *d*(T) for the *b*-ZnO NPs suggests that the use of *b*-EtOH as a reaction solvent promotes a different amount of ligands to adhere to the NPs’ surface or larger surface electrical dipoles [30]. Ink viscosity, *η*, (in cP at 20 °C), can affect the *d*(*H*) values, but, in this case, it remained constant to 3 cP for all the inks (see Table 1) [31].

Thermogravimetric analysis (TGA) was performed on dried synthesized NPs (before redispersion in alcoholic solution and ligands) to determine the number of acetate groups, originating from the reagent zinc acetate, on the nanoparticle surface. Thus, the weight loss on the TGA corresponds to the organic phase (acetate groups) surrounding the ZnO NPs. As reported in Table 1, a weight loss of 5.2%, 8.9%, and 9.2% was observed for the *b*-ZnO, *r*-ZnO, and Ref-ZnO NPs, respectively. The amount of the organic phase on the ZnO surface has been demonstrated to affect the stability of the resulting NPs [34]. Increasing the number of acetate groups (thus the weight loss), creating chelating or bridging-type structures on the ZnO surfaces, is expected to stabilize the resulting suspensions. These results suggest an improved stability of the *r*-ZnO ink (similar to the Ref-ZnO) compared to *b*-ZnO, highlighting a dependency between the reaction solvent and the number of exposed acetate groups.

Based on results reported above, the use of *b*-EtOH and *r*-MeOH as alternative and more sustainable reaction solvents generates ZnO NPs with chemical/physical properties comparable to those of Ref-ZnO, prepared using non-recycled MeOH. Note that, despite the general narrow variation in NP size (Table 2), the observed differences might be ascribed to the reaction solvent that, being the only variable during the preparation of the ZnO NPs, is likely to influence the growth and thus the dimensions of the resulting nanomaterials. The final ZnO NP inks have the same viscosity, allowing for the preparation of high-quality thin-films with common and scalable solution-processing techniques such as blade coating, slot-die coating and ink-jet printing [9,35].

### 3.2. ZnO Thin-Films

Thin-films were prepared in air by depositing ZnO NP inks on glass/ITO substrates by blade coating, a reproducible and scalable lab-scale deposition technique [36]. To reproduce the operating conditions, NP inks were deposited on the top of O_2_ plasma-treated glass/ITO substrates and then thermally annealed using the same processing conditions adopted for the preparation of optimized OSCs (see Section 2 for details). The thickness of the resulting films, measured using a profilometer, was ≈50 nm.

Hereafter, we compare and discuss the optical, morphological, and electronic properties of the new ZnO inks versus the reference system, Ref-ZnO.

**Figure 2:**
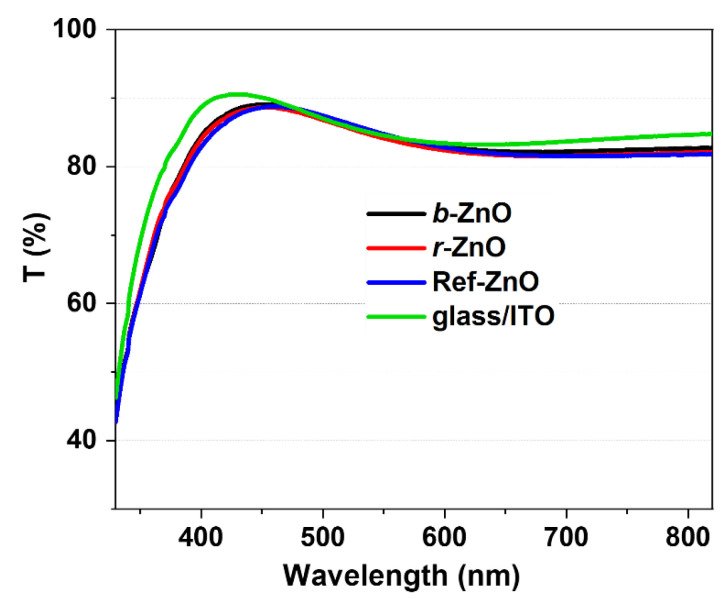
Optical transmission spectra of the different ZnO NP thin-films deposited on glass/ITO substrates.

The optical transparency of the thin-films was investigated by spectrophotometric analysis. Figure 2 shows the optical transmission spectra of the different glass/ITO/ZnO sub-structures compared to pristine glass/ITO. The resulting spectra of the different structures based on the *b*-ZnO, *r*-ZnO, and Ref-ZnO NP inks are identical with a slight reduction in the transmission in the blue region (from 330 nm to 450 nm) compared to glass/ITO (green curve in Figure 2). This result confirms the transparency of all the ZnO films over the entire visible range.

FTIR analysis (ATR techniques) was also performed on the ZnO thin-films to investigate the absence of possible contaminants due to the use of different solvents (e.g., bioethanol and *r*-MeOH). Figure S3 shows the ATR spectra of the different ZnO films before and after thermal treatment, characterized by three main signals at (i) ≈550 cm^−1^, typical of Zn-O vibrations [37]; (ii) ≈830 cm^−1^, relative to the glass/ITO substrate; and (iii) ≈1420–1580 cm^−1^, likely due to the COO-Zn stretching modes [38]. As evidenced by the nearly identical spectra, we can confirm the absence of relative differences or specific features ascribable to possible contaminations (even volatile) during the synthesis step.

**Figure 3:**
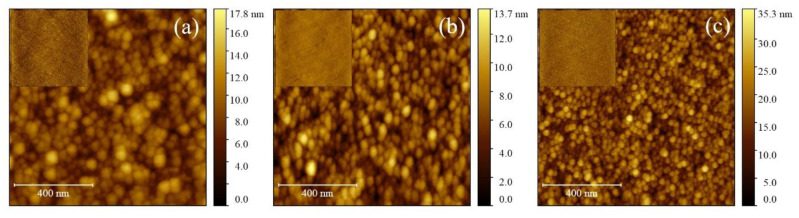
AFM images, 1 × 1 μm^2^, of the *b*-ZnO (**a**), *r*-ZnO, (**b**) and Ref-ZnO (**c**) thin-films. Insets are 30 × 30 μm^2^ images of the same samples at a lower magnification to investigate film homogeneity.

The surface nanomorphology of blade-coated ZnO NP layers were investigated using atomic force microscopy (AFM). Figure 3 shows the resulting topographic images with high magnification, 1 × 1 μm^2^, of the *b*-ZnO (Figure 3a), *r*-ZnO (Figure 3b), and Ref-ZnO (Figure 3c) layers. The insets show the corresponding surface morphology on a larger area: 30 × 30 μm^2^.

All ZnO NP films homogeneously cover the glass/ITO substrates, displaying compact, featureless, and defect-free surfaces at a larger scale-length, as confirmed by the insets (30 × 30 μm^2^). These observations were confirmed by the RMS surface roughness, σ, that was always lower than 5 nm. Specifically, σ was ≈2.9 ± 0.2, 2.4 ± 0.1, and 4.4 ± 0.4 nm for *b*-ZnO, *r*-ZnO, and Ref-ZnO, respectively.

Despite the film flatness, the higher-magnification AFM images, with a scale-length of 1 × 1 μm^2^, show a homogeneous distribution of NPs with a progressive decrease in NP size passing from *b*-ZnO (bigger NPs in diameter, Figure 3a) to Ref-ZnO (smaller NPs in diameter, Figure 3c). By adopting the image analysis reported by Portale et al. [39], the average diameter *d(A)* of the NPs for each sample was estimated to be 32.8 ± 0.8, 17.9 ± 0.5, and 12.5 ± 0.3 nm for *b*-ZnO, *r*-ZnO, and Ref-ZnO, respectively. Note that, *d*(A) measured using AFM is larger than *d*(H) and *d*(T) due to tip convolution effects [40] and NP organization on a (relative) rough surface [41]. To compare *d*(A) measured from the films to *d*(H) measured in solution, as well as *d*(T) measured from highly dispersed NPs on a TEM grid, tip convolution effects can be removed by surface reconstruction through computational modelling, but this is out of the scope of this manuscript. However, the trend of *d*(A) is the same when observed in the DLS and TEM analyses.

**Figure 4:**
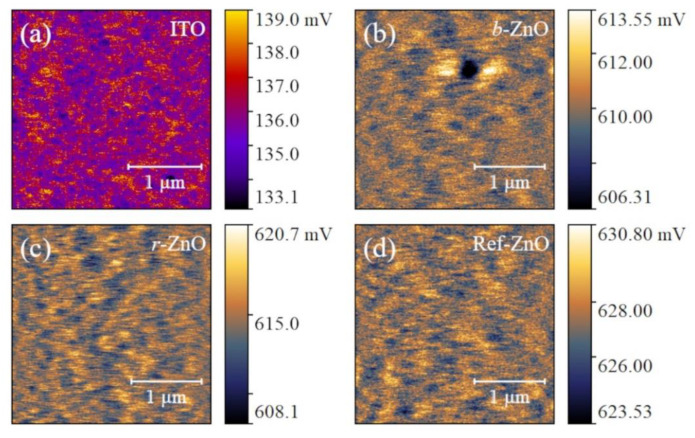
KPFM images of the ITO surface (**a**) and of the *b*-ZnO (**b**), *r*-ZnO, (**c**) and Ref-ZnO (**d**) thin-films.

Kelvin Probe Force Microscopy (KPFM) measurements were performed on glass/ITO/ZnO sub-structures to determine and compare the contact potential difference (V_CPD_) for the *b*-ZnO, *r*-ZnO, and Ref-ZnO NPs with respect to the glass/ITO substrate (Figure 4).

KPFM measurements were performed using the second pass technique, where the first pass acquires the topography and the second retracts the tip 40 nm away from the surface, keeping the distance constant along the scan [42]. The V_CPD_ measured using KPFM on both the glass/ITO and glass/ITO/ZnO sub-structures showed surface potential values with a Gaussian distribution. For the glass/ITO structure (Figure 4a), the average value of the V_CPD_ was (136 ± 1) mV, while it increased to ≈ 615 mV for the glass/ITO/ZnO structures. Specifically, the V_CPD_ slightly increased for decreasing NP diameters, i.e., (614 ± 2) mV, for the *b*-ZnO, *r*-ZnO and Ref-ZnO NPs (see Figure 4b,c), even though a variation of ≈3% can be considered negligible.

Briefly, KPFM works like a Kelvin Probe (see the Supplementary Materials in ref. [43]); if an external electrical contact is made between the two electrodes, i.e., the cantilever tip (in Pt) and the conductive (or semiconductive) substrate, their Fermi levels equalize and the resulting movement of electrons from the metal flow with a lower work function (WF) to produces a V_CPD_.

If the WF of the tip Φ_T_ is known (in our case, it is a polycrystalline Pt film with a WF = (5.30 ± 0.07) eV) [44], then the WF of the sample Φ_S_ can be calculated from the measured V_CPD_ (measured in V) by nullifying the AC component of the electrostatic force between the tip and the sample [45]:

Φ_S_ = Φ_T_ + V_CPD_ (1)

Thus,

V_CPD_ (in V) = Φ_S_ (in eV) − Φ_T_ (in eV) = Φ_S_ − Φ_T_ / (– e) = Φ_T_ (in V) − Φ_S_ (in V) (2)

As explained in ref. [42], the general form of Φ valid for metals, insulators, and semiconductors is the sum of four potentials: (i) the distance between the Fermi level and the bottom of the conduction band in the bulk; (ii) the energy band curvature on the surface due to surface states; (iii) the electron affinity energy with the lattice; and (iv) the dipole component determining the potential drop due to molecules adsorbed onto the surface.

ITO is a highly degenerate n-type semiconductor or, alternatively, a low-carrier-concentration metal. In our experiments, ITO [46] had a resistivity of ≈2·10^−4^ Ω·cm with a volume charge concentration of 10^21^ cm^−3^; hence, the fermi level of ITO is within the conduction band [47]. In a highly degenerate semiconductor, the energy band curvature is null [48], and the distance between the Fermi level and the bottom of the conduction band is as described above. Accordingly, the ITO WF, Φ_ITO_, is

Φ_ITO_ = Φ_bulk-ITO_ + V_D-ITO_ (3)

where Φ_bulk-ITO_ is the bulk work function of ITO, spanning the range (4.67–4.83) eV, as measured using photoelectron spectroscopy and KPFM [49], while the V_D-ITO_ is the potential drop due to molecules adsorbed onto the ITO surface.

Similarly, the work function of the metal tip, Φ_T_, is

Φ_T_ = Φ_bulk-T_ + V_D-T_ (4)

By combining Equations (3) and (4), the V_CPD_ measured using KPFM is

V_CPD_ (in V) = Φ_T_ (V) − Φ_S_ (V) = Φ_bulk-T_ + V_D-T_ − Φ_bulk-ITO_– V_D-ITO_ = (Φ_bulk-T_ − Φ_bulk-ITO_) + (V_D-T_ − V_D-ITO_) = (Φ_bulk-T_ − Φ_bulk-ITO_) + A (5)

where the term A includes the voltage drops due to molecular contaminations on the tip and sample. By averaging the WF data intervals reported above from the literature, i.e., (4.75 ± 0.08) eV, chemically controlled surfaces of ITO should have a theoretical V_CPD_, V_CPD-Th_, of (Φ_bulk-T_ − Φ_bulk-ITO_) = 5.3 − 4.75 = 550 mV with a relative error of ≈3%, and thus V_CPD-Th_ is expected to be (550 ± 20) mV. As reported above, V_CPD_ measured using KPFM on a glass/ITO structure was (136 ± 1) mV. The voltage drop A, due to an adsorbate layer on the tip and ITO surfaces, is calculated using Equation (5) as A = V_CPD_ − V_CPD-Th_ = 136 − 550 = −414 mV, i.e., −(414 ± 18) mV, considering its absolute error or (414 ± 18) meV when expressed in eV. This result agrees with the ≈400 meV of ITO when stabilized by airborne contaminants after O_2_ plasma cleaning [50], and therefore it sets a voltage offset of the V_CPD_ for KPFM measurements when performed in air.

The KPFM measurements on the structure glass/ITO/ZnO NP films have a homogeneous V_CPD_ ≈ 615 mV, in detail (615 ± 9) mV, since V_CPD_ is (610 ± 1), (614 ± 2), and (627 ± 2) mV for b-ZnO, r-ZnO, and Ref-ZnO, respectively. By using Equation (5) and A = −(414 ± 18) mV, (Φ_bulk-T_ − Φ_bulk-ZnO_) = V_CPD_ − A and so the difference between the WF of the Pt-coated tip and the ZnO samples is equal to (1029 ± 62) mV, approximated to (1030 ± 60) mV, i.e., (1.03 ± 0.06) V or −(1.03 ± 0.06) eV.

**Figure 5:**
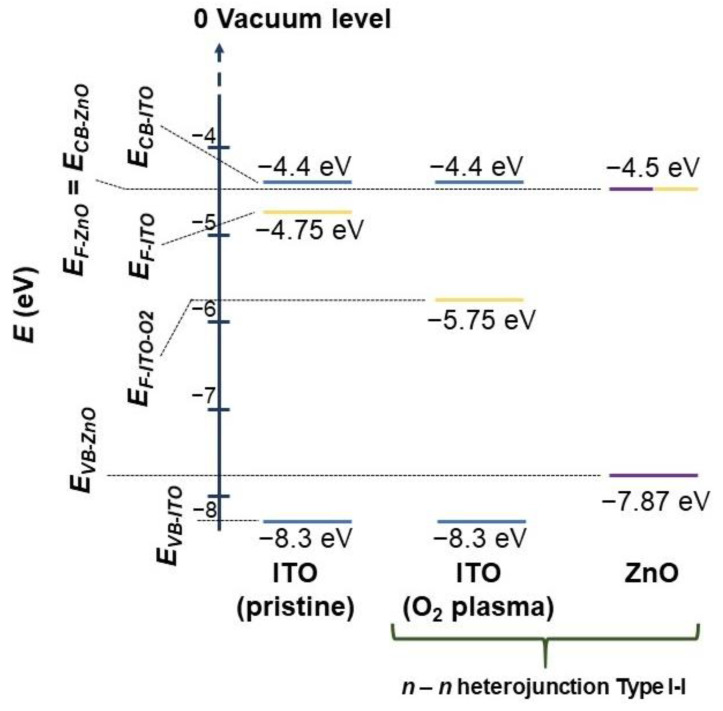
Band diagram of the glass/ITO/ZnO NP thin-films using KPFM measurements. The ITO WF is (4.75 ± 0.08) eV, obtained by averaging the maximum, 4.83 eV, and the minimum, 4.67 eV, values in the literature. The absolute error is the discrepancy, i.e., (4.83–4.67)/2. The bottom of the conduction band (*E_CB_*) of the ITO is 4.4 eV [53], while the bang gap *E_G_* is in the range [3.5, 4.3] eV [54], i.e., on average (3.9 ± 0.4) eV. The WF and *E_CB_* are considered equal to 4.5 eV [55], whereas the *E_G_* ≈ 3.37 eV [56], which brings the top of valence band to *E_VB_* ≈ 7.87 eV (all energy values in the band diagram are negative since the vacuum level is fixed to zero).

In view of these results, the ITO and ZnO film form an *n*−*n* heterojunction of Type I-I (see band diagram in Figure 5) [51,52]. Since the Fermi levels were aligned when the *n*−*n* heterojunction was formed, an additional surface potential of ≈1.25 eV was observed using KPFM due to the different Fermi levels. The bulk WF Φ_bulk-ZnO_ calculated from KPFM passes from 6.33 to 5.08 eV and then to ≈4.66 eV by removing the voltage drop due to molecular contamination.

Since Φ_bulk-T_ = (5.30 ± 0.07) eV, −Φ_bulk-ZnO_ = Φ_bulk-T_ − (V_CPD_ − A) = 5.30 + 1.03 = 6.33 eV with the absolute error Φ_bulk-ZnO_ = (6.33 ± 0.45) eV. As reported in the literature [57,58], Φ_bulk-ZnO_ for a ZnO film is considered the same as the electron affinity, i.e., ≈4.5 eV [55], which is ≈1.8 eV lower than the Φ_bulk-ZnO_ calculated from KPFM. Such an energy difference cannot be ascribable to the voltage drop due to molecular contaminations of the tip and sample, so KPFM contrast is sensitive to the whole ITO/ZnO structure not only the facing ZnO film. Indeed, the ITO was cleaned by O_2_ plasma prior to ZnO deposition (few minutes after), and Φ_bulk-ITO_ increased by ≈1 eV, passing from 4.75 to 5.75 eV [50]. Concurrently, airborne contaminants were removed at the ITO–ZnO interface; hence, A ≈ 0, although contaminants are present at the tip−ZnO interface.

This value is very close to the bulk one (≈4.5 eV) reported in the literature, and since the metal tip causes a small interfacial band bending at the ZnO surface [59], the literature and measured Φ_bulk-ZnO_ of the ZnO film are near-identical [57,58]. In conclusion, ZnO materials have suitable WFs, providing well-matched contact energetics with the adjacent layers (LUMO of the acceptor materials of the active blend and ITO) within an inverted OSC, thus favoring the electron extraction process.

### 3.3. Photovoltaic Devices

To test the electrical performance of the ZnO NP thin-films, a set of inverted bulk heterojunction (BHJ) solar cells with structure glass/ITO/ZnO/PTB7:PC_71_BM (1:1.5 *wt*/*wt*)/MoO_x_/Ag was fabricated and characterized. Additional details on the materials, processing conditions, and characterization are reported in Section 2.

**Figure 6:**
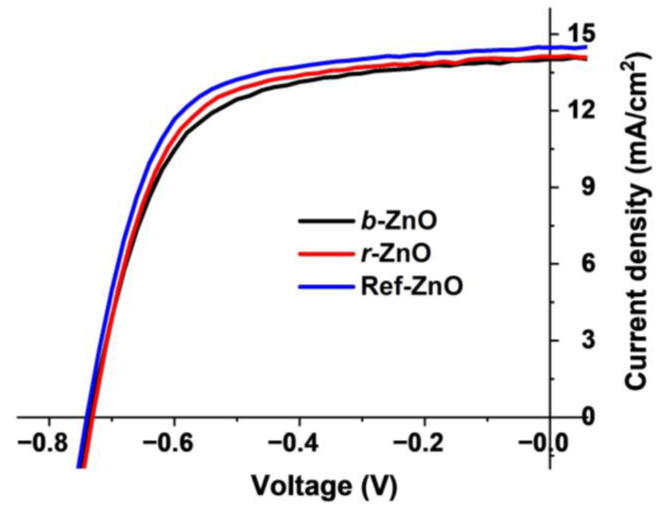
J-V curves of the inverted PTB7:PC_71_BM solar cells based on the different ZnO NP thin-films as an ETL.

Table 2 summarizes the photovoltaic responses of the resulting devices, including the open-circuit voltage (V_OC_), short-circuit current density (J_SC_), fill factor (FF), and PCE. Figure 6 shows the corresponding current density–voltage (J-V) curves. The photovoltaic performance of the resulting PTB7:PC_71_BM-based solar cells, consistent with analogous systems reported in the literature [16,22,23], are comparable, giving PCE values between 6.5 and 6.9%. This confirms the general quality and suitability of all the NP inks/films for photovoltaic applications, regardless of the adopted reaction solvent. Nevertheless, despite the satisfactory photovoltaic performance of both the *b*-ZnO- and *r*-ZnO-NP-based devices, containing *r*-ZnO seems to work slightly better than the analogous systems with *b*-ZnO, thus giving results nearly identical to the reference system (Ref-ZnO). Except for typical fluctuations of the solution-processed devices, all the cells exhibit comparable V_OC_ values reflecting the same WF values observed for the ZnO NPs. The main difference was represented by the FF, which passes from 63 to 66% for the *b*-ZnO- and *r*-ZnO-based devices, respectively, while a slightly improved Jsc was observed for the reference system (Ref-ZnO). Notably, the observed differences were solely ascribed to the ZnO layer and its adjacent interfaces, being the only difference within the studied devices.

As previously discussed, all ZnO NP films showed nearly identical optical and electronic properties except for some differences at the morphological level. Looking at the photovoltaic results, we found a correlation between the NP dimensions (Ref-ZnO < *r*-ZnO < *b*-ZnO), determined by DLS, TEM and AFM, and the corresponding device performance (Ref-ZnO > *r*-ZnO > *b*-ZnO). When increasing the NP dimensions, we observed a decrease in device efficiency (from 6.9% to 6.5%).

**Table 2:** Photovoltaic responses of the inverted PTB7:PC_71_BM solar cells based on the different ZnO NP thin-films as an ETL. The reported values are the averages of five different devices.

NPs as the ETL	V_OC_ (V)	J_SC_ (mA/cm^2^)	FF (%)	PCE (%)
*b*-ZnO	0.733 ± 0.003	14.0 ± 0.3	63 ± 1	6.5 ± 0.2
*r*-ZnO	0.731 ± 0.010	14.1 ± 0.4	66 ± 2	6.8 ± 0.2
Ref-ZnO	0.738 ± 0.004	14.5 ± 0.4	65 ± 1	6.9 ± 0.2

This trend might be determined by a slight increase in the internal resistance at the ZnO–BHJ interface, affecting the FF and/or Jsc values. Indeed, the different dimensions of the NPs, correlated with an increased surface roughness of the films (Figure 3), might be responsible of the following effects: (i) a change in the nanoscale surface quality (and contact resistance) at the ZnO–BHJ (or ITO–ZnO) interface, responsible for charge extraction processes; and (ii) a variation in the NP interactions within the ZnO bulk, responsible for charge transport processes [6,60]. Thus, the larger the NP dimensions and surface roughness of the films, the larger the contact resistance and the lower the efficiency of the related charge extraction/transport processes.

In view of a possible use of these inks on flexible and/or bio-based substrates [16], we investigated the effect of lower thermal treatments of the ZnO NP layers on the resulting device performance. To this end, instead of a thermal annealing at 120 °C for 10 min, used for the devices reported in Table 2 and Figure 6, we treated the ZnO films at 85 °C for 20 min. The photovoltaic responses and the trend of the resulting devices (Table S1 and Figure S4) were comparable to those obtained with stronger thermal treatments (Table 1), confirming the quality of the ETL even if treated at lower temperatures.

Finally, our results not only demonstrate the excellent optical, morphological, electronic, and electrical properties of *b*-ZnO and *r*-ZnO NPs but also validate the effectiveness of a more sustainable synthetic approach, providing a contribution for the development of alternative methods and materials for the next generation of eco-designed systems.

## 4. Conclusions

In this work, we investigated the effect of different reaction solvents used for the synthesis of ZnO NPs to be employed as an electron-transporting layer in organic solar cells. We studied and compared three different ZnO NPs obtained from (i) recycled methanol (*r*-ZnO), (ii) bioethanol (*b*-EtOH), and (iii) commercial methanol as the reference system (Ref-ZnO).

The effect of the solvents on the optical, morphological, and electronic properties of the resulting ZnO NPs, both in solution and thin-films, was investigated and discussed. In general, we found that all NPs had nearly identical properties independently from the reaction solvent. The only difference was represented by a slight variation in the NP dimensions, as confirmed by the UV–vis, DLS, AFM and TEM analyses. The hydrodynamic diameters (*d*(*H*), Table 1) estimated for the inks were found to be ≈ 14, 7, and 6 nm for the *b*-ZnO, *r*-ZnO, and Ref-ZnO NPs, respectively, following the same trend observed with other techniques.

To further investigate the properties of the ZnO NPs, we fabricated a set of inverted PTB7:PC_71_BM-based OSCs containing ZnO NPs as an ETL. PCEs of 6.5 and 6.8%, comparable to the reference system and consistent with analogous devices reported in the literature, were obtained for *b*-ZnO and *r*-ZnO, respectively, highlighting the quality and suitable properties of the reported materials.

These results demonstrate that the use of *b*-EtOH and *r*-MeOH as alternative and more sustainable reaction solvents generate ZnO NPs with chemical/physical properties, in solution and in a solid state, comparable to the reference system. This confirms the efficacy of the proposed approach, providing a relevant contribution for the development of more sustainable and scalable methods/materials towards the next generation of green electronics.

## Figures and Tables

**Table 1:** Characteristics of the different ZnO NP inks.

NPs	λ*_sh_* (nm)	Hydrodynamic Diameter (*d(H)*) (nm)		Viscosity at 20 °C (cP)	Weight Loss ^(a,b)^ (%)
		After Synthesis ^(a)^	Final Ink		
*b*-ZnO	350	15.8 ± 1.2	14.5 ± 1.2	3 ± 1	5.2 ± 0.2
*r*-ZnO	338	11.0 ± 1.3	7.2 ± 1.3	3 ± 1	8.9 ± 0.3
Ref-ZnO	333	19.5 ± 1.3	6.4 ± 1.3	3 ± 1	9.2 ± 0.3

^(a)^ determined after NP synthesis, before redispersion in the commercial alcohol and ligand; ^(b)^ the relative error in the weight loss was fixed at 3%, as reported in refs. [32,33].

## Data Availability

Data are contained within the article and Supplementary Materials.

## Supplementary Materials

The following supporting information can be downloaded at https://www.mdpi.com/article/10.3390/ma17061332/s1. Figure S1: DLS plots of all the ZnO NPs after synthesis. Figure S2: TEM images of the NPs. Ten cross-section profiles marked by using the software Gwyddion (version 2.65) are reported in the figure. Details of the analysis of NP diameter are described. Figure S3: ATR spectra of the blade-coated ZnO thin-films on glass/ITO, before and after thermal treatment: (a) no thermal treatment, (b) 85 °C for 20 min, and (c) 120 °C for 10 min. Figure S4: J-V curves of the inverted PTB7:PC_71_BM solar cells based on the different ZnO NP inks thermally annealed at 85 °C for 20 min. Table S1: Photovoltaic responses of the best performing inverted PTB7:PC_71_BM solar cells based on the different ZnO NP films, thermally annealed at 85 °C for 20 min.
