# Supplementary material for: Recycled or Bio-Based Solvents for the Synthesis of ZnO Nanoparticles: Characterization and Validation in Organic Solar Cells
Source: Materials (Basel). 2024 Mar 14;17(6):1332. doi: 10.3390/ma17061332 (PMC10971963; doi:10.3390/ma17061332)
Supplement: Supplementary file 1 [file materials-17-01332-s001.zip › materials-2893935-supplementary.pdf]

Supporting Information

Recycled or Bio-Based Solvents for the Synthesis of ZnO Nanoparticles: Characterization and Validation in Organic Solar Cells

*Cristiano Albonetti [1], Riva Alkarsifi [2], Virginie El Qacemi [2], Benjamin Dhuiege [2], Giampiero Ruani [1], Mirko Seri [1,]**

[1] National Research Council (CNR) - Institute of Nanostructured Materials (ISMN), Via P. Gobetti 101, 40129-Bologna, Italy.

[2] GenesInk, 39 Avenue Gaston Imbert, 13790 Rousset, France

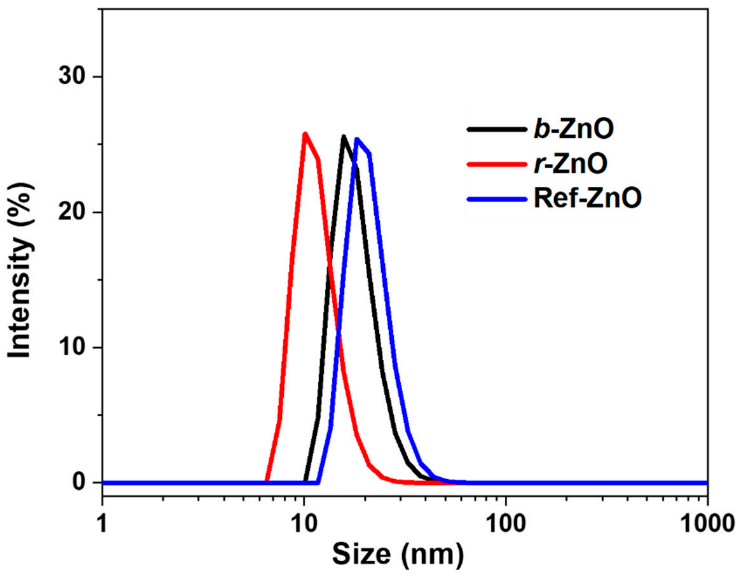

Figure S1. DLS plots of all the ZnO NPs after the synthesis (before redispersion in the commercial alcohol and ligand).

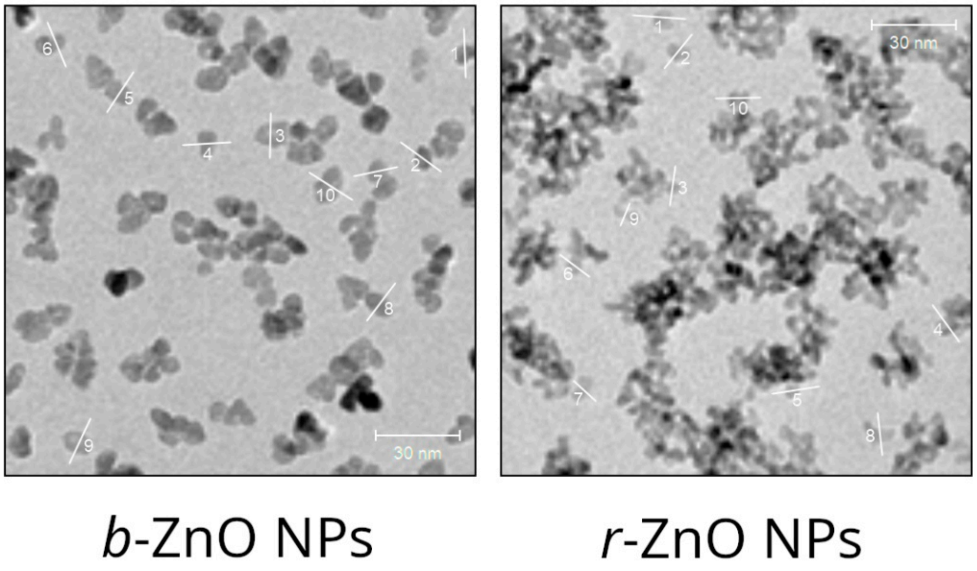

Figure S2. TEM images of NPs. Ten cross-section profiles marked by using the software Gwyddion are reported in the figure (white numbered lines). Details on the analysis of the NP diameter are described below.

NPs diameter measured by TEM

The diameter of *b*-ZnO and *r*-ZnO NPs were measured form TEM images (see Figure S2). By using the software Gwyddion (*Nečas, D., & Klapetek, P. (2012). Gwyddion: an open-source software for SPM data analysis. Central European Journal of Physics, 10(1), 181–188. https://doi.org/10.2478/s11534-011-0096-2*), ten cross-section profiles are traced across single NPs

(see white lines in Figure S2). Each single profile is fitted by negative step-height function for measuring the step width, i.e. the NP diameter $d(T)$. Lastly, $d(T)$ values are averaged to obtain the mean $d(T)$ with an absolute error calculated from discrepancy, i.e. $(d(T)_{max} - d(T)_{min})/2$. In average, $d(T)$ is (5 ± 1) and (6 ± 1) nm for *b*-ZnO and *r*-ZnO NPs, respectively.

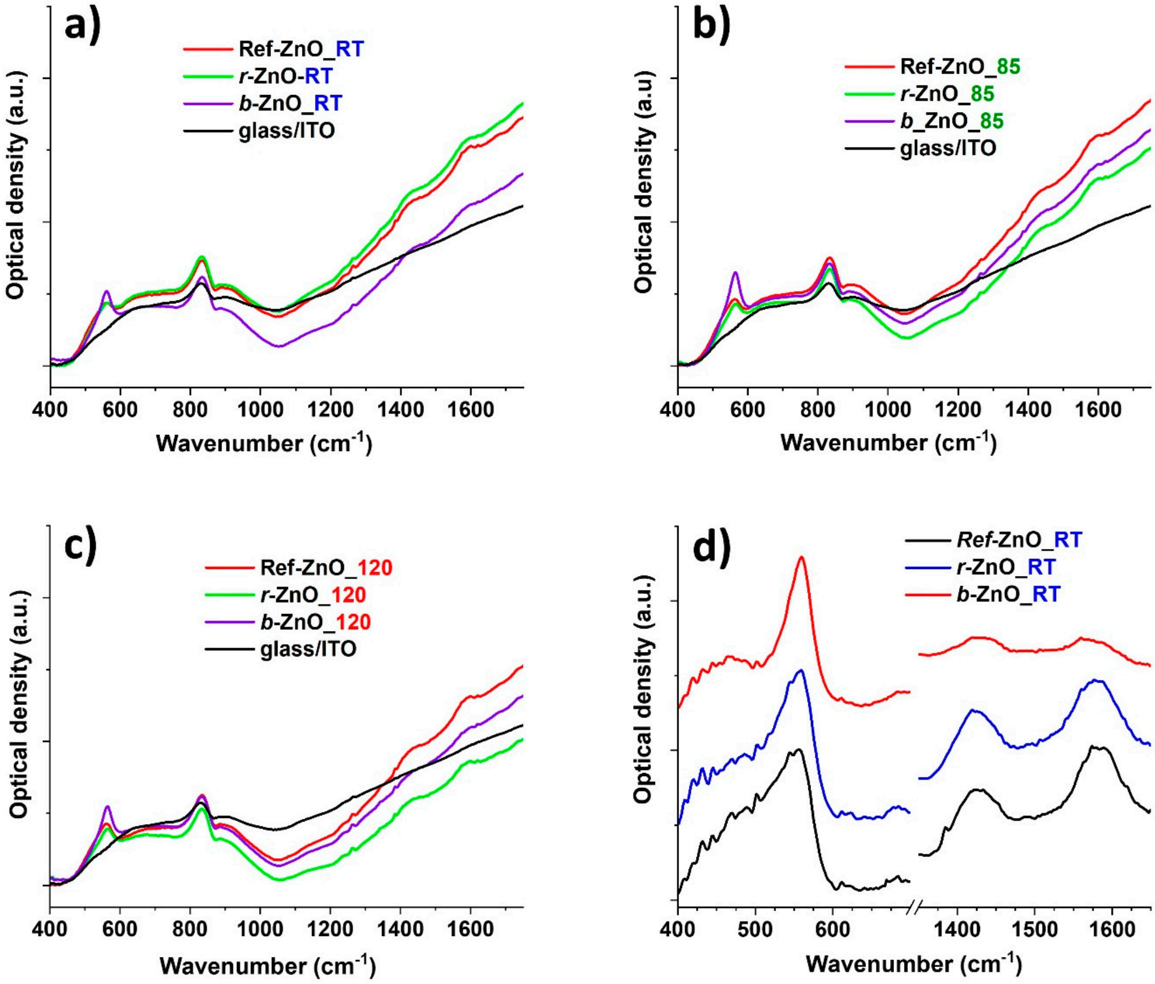

Figure S3. ATR spectra of the ZnO thin-films blade-coated on glass/ITO, before and after thermal treatments: (a) no thermal treatment, (b) 85°C for 20 minutes and, (c) 120°C for 10 minutes. Figure (d) shows the ATR spectra of the three different ZnO NPs before thermal treatments. The spectra have been corrected for the substrate contribution and normalized to the intensity of the peak at 558 cm-1. Interestingly, *b*-ZnO shows a much less intense bands associated with COO-Zn (1420 and 1580 cm^{-1}) likely due to the smaller surface/volume ratio (ascribable to the larger dimension of the b-ZnO particles compared to the others).

Table S1. Photovoltaic responses of best performing inverted PTB7:$PC_{71}BM$ solar cells based on the different ZnO NP films, thermally annealed at 85°C for 20 minutes.

**ZnO**	**V_{OC}** (V)	**J_{SC}** (mA/cm^2)	**FF** (%)	**PCE** (%)
b-ZnO	0.70	15.0	61	6.4
r-ZnO	0.71	15.6	63	7.0
Ref-ZnO	0.73	14.2	66	6.9

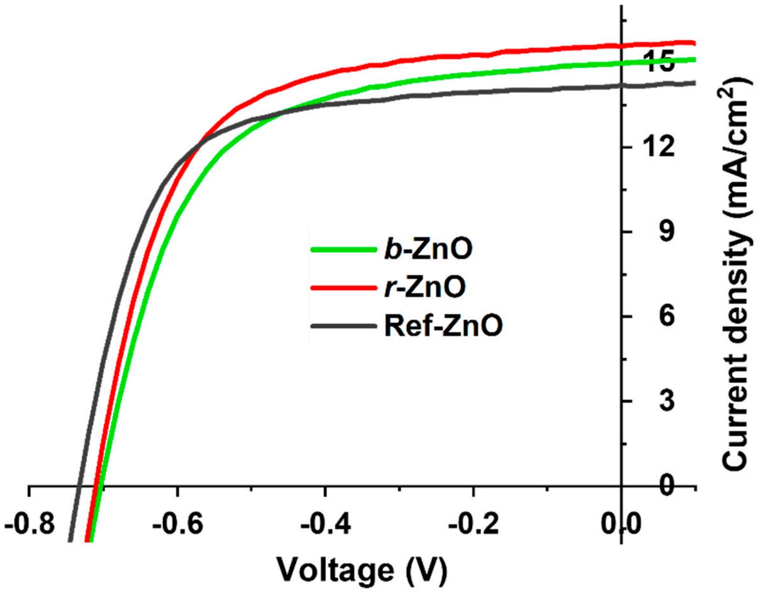

Figure S4. J-V curves of inverted PTB7:$PC_{71}BM$ solar cells based on different ZnO NPs inks thermally annealed at 85°C for 20 minutes.
